# Investigation of Content Parameters in Wet-Fractionated Fibre from Various Plants for Potential Use in Human Nutrition

**DOI:** 10.3390/foods11193038

**Published:** 2022-09-30

**Authors:** Gábor Csatári, Bence József Eged, Csaba Fehér, Miklós Gábor Fári, Szilvia Kovács

**Affiliations:** 1 Department of Applied Plant Biology, Institute of Crop Sciences, University of Debrecen, Böszörményi Street 138, 4032 Debrecen, Hungary; 2 Department of Applied Biotechnology and Food Science, Budapest University of Technology and Economics, Szent Gellért tér 4, 1111 Budapest, Hungary

**Keywords:** fibre, glucan, xylan, arabinan, Klason lignin, total phenol, total flavonoids, total protein, alfalfa, broccoli, soy

## Abstract

Green biorefining uses fresh lignocellulosic biomass to produce green juice and pressed fibre fractions by wet fractionation. The latter is a byproduct, accounting for 25–32% of the starting material. In this study, the composition (glucan, xylan, arabinan, lignin, total phenol, flavonoid and protein) of pressed fibres obtained from four alfalfa, four soy and one broccoli varieties were determined at different harvest times. Statistical analyses were performed to determine the effects of harvest time and variety on the measured parameters. In most of the cases, there were interactions between the effects of harvest time and variety. Among alfalfa varieties, OLI1 had the highest carbohydrate (52.09 *w/w*%) and DIM3 had the lowest lignin (13.02 *w/w*%) content. In the case of soy, the ADV2 variety had the highest carbohydrate (53.47 *w/w*%) and PK1 had the lowest lignin (11.14 *w/w*%) content. Broccoli contained low amounts of carbohydrates (44.94 *w/w*%) and lignin (10.16 *w/w*%). The phenolic and flavonoid contents were similar for each species, but the protein content was the highest in alfalfa fibre. Based on these data, the most promising species, varieties and harvesting time can be selected in terms of a certain component that could be essential to produce functional foods with enhanced nutritional value.

## 1. Introduction

Green biomass is a very good source of renewable raw material for green biorefining [1]. Its most common component is lignocellulose that is derived from the plant cell wall [2]. Green biorefining is a complex system that aims to apply sustainable, environmentally and resource-friendly technology. The aim is to use all the biomass components generated, from which to produce bioenergy and high-value-added products [3]. In green biorefining, the green biomass is pressed to produce green juice and press fibre residue. The resulting fractions contain a wide range of compounds that make them valuable [4]. The global produced lignocellulosic biomass is more than 180 billion tonnes annually [5]. Lignocellulose mainly consists of cellulose, hemicellulose and lignin [6]. Lignin can be divided into acid-soluble and acid-insoluble parts, depending on its solubility during acidic biomass treatments [7,8]. The three components of lignocellulose (cellulose, hemicellulose and lignin) belong to the group of water-insoluble dietary fibres, from a human nutritional point of view, which have effects on human health such as increasing faecal bulk and reducing intestinal transit time [9,10,11,12,13]. In addition to cellulose, hemicellulose and lignin, the cell wall also contains other compounds that play an important role in human health, such as proteins and phenols [14], and their quantity, quality and properties also depend on the type of fibre [14,15].

Today, the circular economy is an important element in the sustainable use of natural resources. The basic concept is to extend the life cycle of products, either by recycling them or by turning them into higher-added-value products. This is a necessity, as the growth of the world’s population and increasing climate change also justify the optimal use of biomass produced by agriculture [16,17,18]. This also raises economic issues, as cost efficiency and sustainability may be enhanced [19,20]. The concept of green biorefining is an excellent fit with a circular economy; however, the byproducts of wet fractionation (e.g., press fibre) are often undervalued, despite the fact that they contain high-value components [21,22]. The possible uses of pressed fibre are extremely varied. It can be an excellent raw material for biogas production, biofuel production and can also be used as a raw material for many other industrial applications, such as pharmaceuticals and paper [4]. It is suitable for feeding ruminants because of its excellent nutritional value, in particular, its protein content and its amino acid composition [21,23]. Pressed fibre can also be an excellent substrate for growing oyster mushrooms (*Pleorotus ostreatus*), as evidenced by Zhou et al. [24]. Some of the components of lignocellulose can also be used in the food industry, e.g., xylose and arabinose as natural sweeteners [25,26]. It also contains other phytonutrients important for the human body such as phenols [27,28], as well as the proteins mentioned earlier [21]. A key objective would be to buy back lignocellulosic substrates from farmers, increasing the role of biorefineries in the circular economy [29].

Extensive knowledge about the yield and composition of pressed fibres originated from different sources is essential to develop their circular use and especially for their use in the food industry, e.g., potentially for the production of functional foods. Hence, one of the objectives of this study was to determine the yields of pressed fibres at different harvest times for different varieties of three model plants: alfalfa, soybean and broccoli. The other objective was to quantify the lignocellulosic components (cellulose, hemicellulose and lignin) and protein, as well as the total phenol and flavonoid content of the model plants. The current study aims to provide a basis for the future valorisation of the analysed byproducts.

## 2. Materials and Methods

### 2.1. Source of Biomass

All the examined plants were produced in open fields in the experimental site of the University of Debrecen. The field experiment for each variety was set in three replications. We applied general agronomic practice like irrigation, weed control and fertilisation in the production of these crops.

#### 2.1.1. Alfalfa

Alfalfa seeds at 25 kg/ha were sown in March 2018 in chernozem soil. the varieties used in this study were *Danubia*, *Dimitra*, *Plato* and *Olimpia*. The varieties were set in three repetitions. The plot size was 10.0 m^2^, with a randomised block layout. The dates of the alfalfa harvest were June, August and October 2018, when the plants were harvested in a green bud state before flowering. The harvest took place in the morning, followed by processing in a laboratory. The quantity of the sample collected from each variety was 1 kg per plot, resulting in 3 kgs of sample for each variety. We referred to the alfalfa varieties involved in the study as Danubia (DAN), Dimitra (DIM), Plato (PLA) and Olimpia (OLI).

#### 2.1.2. Soy

The soy varieties we used were Advisor, Bólyi 612, Isidor and Pannónia kincse. To gain the fresh biomass, the row spacing we used was 24 cm instead of 45 cm, applied in farming practice. Soy seeds at 200 kg/ha were sown. The varieties were set in three repetitions. The size of the plots was 4.52 m^2^, with a randomised block layout. The dates of the soy harvest were June and August 2020. The harvest took place in the morning, followed by processing of the samples in a laboratory. The quantity of the sample collected from each variety was 1 kg per plot, resulting in 3 kgs of sample per variety in total. The plant was cut from the bottom 15 cm of the stem from the soil surface. We referred to the soy varieties involved in the study as Advisor (ADV), Bólyi 612 (BOL), Isidor (ISI) and Pannónia kincse (PK).

#### 2.1.3. Broccoli

Broccoli was planted under field conditions in May 2020. The variety we used was *Calebrese*; according to the farming practice, a 60 × 40 spatial arrangement was used. The harvest took place at the end of May 2020, collecting three specimens. In the case of broccoli, the leaf and petiole were used to study the fibre content.

### 2.2. Fibre Production

Following the harvest of each variety of the model plant species, the green plant biomass was fractionated into green juice and compressed fibre fractions using a twin-screw juicer (Angel Juicer 5500, Angel Ltd., Czech Republic). Samples were stored in a freezer at −21 °C after processing.

### 2.3. Determination of Lignocellulosic Components

#### 2.3.1. Sample Preparation

Before the measurements, the frozen samples were dried at 40 °C for 3 days and the fibre samples were homogenised with a coffee grinder. A modified NREL (National Renewable Energy Laboratory) method was used to determine the fibre components. Based on this information, 0.5 g of dry fibre was added to 2.5 mL of 72% (*w/w*) sulphuric acid. It was kept at room temperature for 2 h, being stirred every 30 min. Distilled water (75 mL) was added and the suspension was placed in an autoclave at 121 °C for 1 h. It was then filtered through a preweighed G4 glass filter with a vacuum pump. The supernatant was filtered through a 45 μm nylon filter [8].

#### 2.3.2. Recoverable Sugars

The supernatatant was used to determine glucose, xylose and arabinose by high-performance liquid chromatography (HPLC) (Detector: Shimazu RID-10 A; Column: BioRad (Hercules, CA, USA) Aminex HPX-87H (300 × 7.8)) at 65 °C. The eluent was 5 mmol/L sulphuric acid, and the flow rate was 0.5 mL/min.

The recoverable sugars measurement was carried out in triplicate. The glucan, xylan and arabinan contents of the fibre samples were determined, taking into account the depolymerisation factor of the monosaccharides [8]. The glucan includes all polysaccharides consisting of glucose monomers.

#### 2.3.3. Klason Lignin

G4 glass filters were placed in a desiccator and weighed after 30 min. Using the modified NREL method, after filtration and washing, the water-insoluble part remains in the glass filter. The glass filter was then placed in an oven at 105 °C overnight and then weighed. The glass filter was then placed in an oven at 550 °C overnight and then weighed. Finally, the Klason lignin content was determined using the appropriate formula [8].

### 2.4. Determination of Total Phenol Content (TPC)

The TPC was determined in the fibre samples according to Singleton and Rossi [30], with minor modifications. A total of 20 mg of the lyophilised powdered fibre sample was placed in 1 mL of methanol:distilled water (70:30), vortexed and incubated in an ultrasonic bath for 30 min. It was then centrifuged at 13,200 rpm for 3 min. The supernatant was filtered through an Eppendorf tube. Subsequently, 1250 µL Folin–Ciocalteu reagent:distilled water (1:10) and 200 µL methanol:distilled water (70:30) were added to 50 µL of the supernatant. After waiting for 1 min, 1000 µL of 0.7 M Na_2_CO_3_ was added and it was incubated in a 50 °C water bath for 5 min. The absorbance was measured at 760 nm (Ultrospec 2100 pro, Amersham BioSciences spectrophotometer). All phenol contents were calculated using a calibration curve, obtained by using the gallic acid standard.

### 2.5. Determination of Total Flavonoids (TFC)

The TFC was determined in the fibre samples according to a modified method of Kim et al. [31]. The determination is based on the complex of flavonol- and flavone-type compounds with aluminium chloride in stoichiometric reaction in acidic medium. The colour intensity of the resulting complex is an indication of the amount of compounds present in the solution. A rutin standard was used to prepare the calibration curve. For the measurement, 0.5 mL of the sample was added to 4.5 mL of Al solution. The Al solution contained 5 mL 10g mL^−1^ AlCl_3_ solution, 5 mL 1 M KOac solution, 75 mL methanol and 140 mL distilled water. The measurement was performed at 415 nm (Ultrospec 2100 pro, Amersham BioSciences spectrophotometer). Results were obtained from the calibration equation in rutin equivalents.

### 2.6. Total Protein (TP)

The TP was determined in the fibre fraction according to the Bradford method [32]. Briefly, 20 mg of lyophilised powdered fibre sample was added to 1 mL of protein solubilising solution, which contained 3.5 M urea, 1 M thiourea, 0.1 M NaOH and 0.6% NaCl, and the pH was 13.00. It was then vortexed for 1 min, incubated in an ultrasonic water bath for 1 h and centrifuged at 10,000 rpm for 5 min. A total of 10 µL of the supernatant was added to an Eppendorf tube and 90 µL of 0.15 M NaCl was added. Then 1 mL of Bradford reagent was added. The absorbance was measured at 595 nm (Ultrospec 2100 pro, Amersham BioSciences spectrophotometer). A standard curve was used to calculate the concentration. The blank was 100 µL NaCl plus 1 mL Bradford reagent.

### 2.7. Statistical Analyses

In the statistical analysis, a two-way ANOVA was used IBM SPSS Statistics 24 (IBM Corp, Armonk, NY, USA), and the effect of two independent variables (variety and harvest) was analysed on the studied parameters (fibre, lignin, glucan, xylan, arabinan, total phenol, total flavonoid and total protein). Before running the ANOVA, a test of normality and Levene’s Test of Equality of Variances were performed. The means were compared by Tukey’s Honestly Significant difference (HSD) test at *p* < 0.05 [33]. In the event that the combined effect of the variables (interaction) was not confirmed, a one-way ANOVA was performed. The explanatory power of the model was characterised by partial Eta squared (Eta^2^).

## 3. Results

### 3.1. Mass of Pressed Fibre from Different Plants

#### 3.1.1. Alfalfa

**Figure 1:**
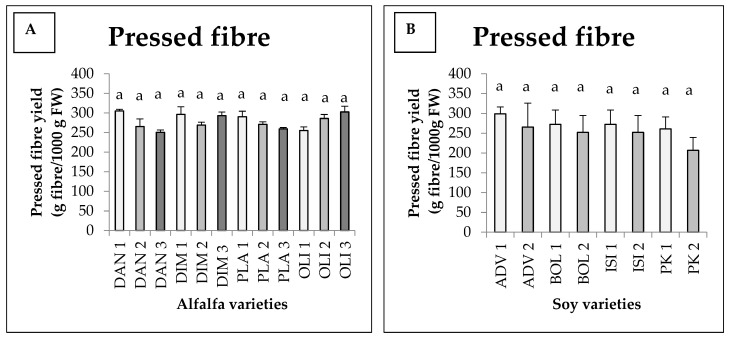
Average press fibre yields of different alfalfa (**A**) and soy (**B**) varieties as a function of harvesting time (+SD). Different letters above the columns refer to significant differences (*p* ≤ 0.05). FW = Fresh weight.

There was an interaction between harvest and variety, jointly affecting the pressed fibre yields; however, there was no statistically verified difference among the pressed fibre yields obtained, considering the interaction (Figure 1A). Nevertheless, the statistical model produced 79% explanatory power (Eta^2^ = 0.788). For the Danubia, Dimitra and Plato varieties, we saw the highest fibre yields in the case of the first harvest, which were 305, 296.33 and 290.33 g, respectively, with the exception of the Olimpia variety, providing its highest figure in the case of the third harvest (302.66 g). For Danubia and Plato, the lowest value was observed during the third harvest (250.66 and 259.33 g). For Dimitra, the lowest value was measured during the second harvest, while for Olimpia, it was measured during the first harvest (Figure 1A).

#### 3.1.2. Soy

From Figure 1B, we can see that for the soy harvests, fibre yields were higher for all varieties in the first harvest. During the first harvest, the highest value obtained is for the Advisor variety (298.66 g) and the lowest is for Pannonia kincse (266.66 g). In the second harvest, a similar trend was observed for each variety. No interaction was found between harvest and variety. Furthermore, there was no significant difference detected between treatments for either variety or harvest.

#### 3.1.3. Broccoli

For broccoli, one harvest date and one variety—the Calebrese variety—was used. The amount of fibre harvested in the case of broccoli was 327.75 g, which is a higher yield of fibre compared to those we obtained from the other two plant species involved.

### 3.2. Klason Lignin and Structural Carbohydrates as a Function of Harvest Time

#### 3.2.1. Alfalfa

**Figure 2:**
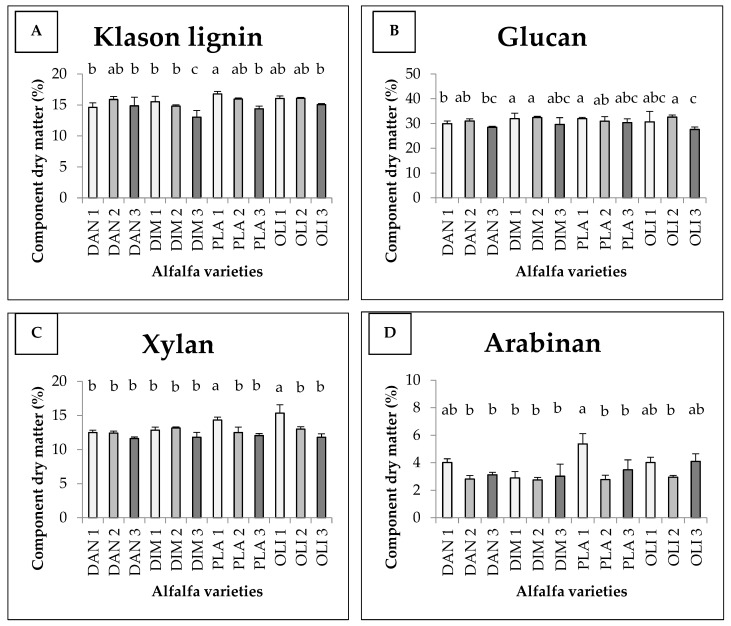
Average Klason lignin (**A**) contents and glucan (**B**), xylan (**C**) and arabinan (**D**) components of different alfalfa varieties as a function of harvest time (+SD). Different letters above the columns refer to significant differences (*p* ≤ 0.05).

The average Klason lignin content and sugar components of different alfalfa varieties are shown in Figure 2. There was an interaction between variety and harvest time, which together determined the quantity of the components under study in the case of lignin, xylan and arabinan. The Klason lignin content is shown in Figure 2A. The Klason lignin content was significantly higher in the first harvest of Plato (16.76 *w/w*%), and significantly different in the third harvest of Dimitra, where the lowest value was measured (13.02 *w/w*%). In six cases (DAN1, DAN3, DIM 1, DIM2, PLA3 and OLI3), the Klason lignin contents had values between the two extremes, being proved statistically different from both. They indicate decreases in the order of the harvests, with the exception of Danubia. The statistical model produced 75% explanatory power (Eta^2^ = 0.746).

The glucan content is shown in Figure 2B. The interaction of the two variables did not affect the glucan content. Although significant differences were detected between harvests, there were no significant differences between varieties. For glucan, the lowest values were measured during the third harvest. The glucan content was 31.95 *w/w*% in the first harvest of Dimitra, 31.95 *w/w*% in the first harvest of Plato and 32.59 *w/w*% in the second harvest of Olimpia, which were the highest values and were significantly detectable. The third harvest of Olimpia was significantly different, with the lowest value (27.60 *w/w*%). We can see the content of xylan in Figure 2C. The statistical model produced 81% explanatory power (Eta^2^ = 0.81). In the case of xylan, the first harvest was the best, with the exception of the Dimitra variety, where the highest value was measured in the second harvest. In the first harvest of Olimpia (OLI1) and Plato (PLA1), significantly higher values of 15.34 *w/w*% and 14.33 *w/w*% were measured compared to the rest of the cases, respectively. Figure 2D shows the content of arabinan. For arabinan, the highest value (5.37 *w/w*%) was measured during the first harvest of Plato (PLA1), which was statistically verified and different from most of the figures displayed, with the exception of DAN1, OLI1 and OLI3.

#### 3.2.2. Soy

**Figure 3:**
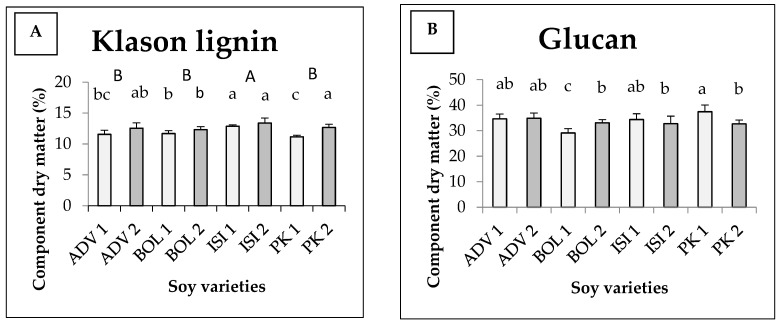

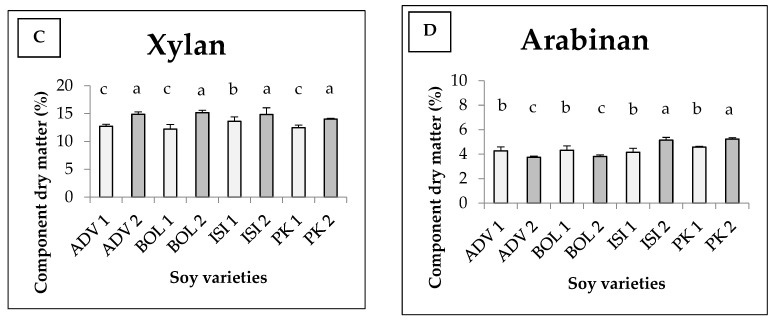
Average Klason lignin (**A**) content and glucan (**B**), xylan (**C**) and arabinan (**D**) components of different soy varieties as a function of harvest time (+SD). Different letters above the columns refer to significant differences (*p* ≤ 0.05). In Figure 3A, the capital letters (A,B) indicate varieties; the normal letters (abc) indicate the harvest.

The average Klason lignin content and sugar components of different soy varieties are shown in Figure 3. There was an interaction between variety and harvesting time, which together determine the quantity of the components under study in the cases of glucan, xylan and arabinan. For Klason lignin (Figure 3A), there was no interaction between the independent variables. The varieties differ significantly, just as the harvests do. A significantly higher Klason lignin content was measured for the Isidor variety, with 12.87 *w/w*% at the first harvest and 13.39 *w/w*% at the second harvest. The later the harvest, the higher the lignin content which can be found in each variety. Figure 3B shows the glucan content. For glucan, the highest value and the lowest value were measured in the first harvest of Pannonia kincse (PK1) (37.42 *w/w*%) and in the first harvest of Bólyi (BOL1) (29.10 *w/w*%), respectively, and they were statistically different from the rest of the values. The statistical model produced medium explanatory power (Eta^2^ = 0.570). The xylan values are shown in Figure 3C. From the results, we can see that in the second harvest we measured a higher value for all varieties. During the first harvest, the highest value was observed for the Isidor variety (13.60 *w/w*%) and the lowest for the Bólyi 612 variety (12.19 *w/w*%). In the second harvest, the Bólyi 612 variety provided the best figure (15.16 *w/w*%), while the worst one was given by Pannónia kincse (14.00 *w/w*%). Figure 3D shows the content of arabinan. Significantly higher values were measured in the second harvest of Isidor and Pannónia kincse. The lowest values were measured in the Advisor and Bólyi second harvests, with statistically significant differences to all the other figures measured, including all the figures measured in each first harvest. Our results show that Pannónia kincse and Isidor are outstanding.

#### 3.2.3. Broccoli

**Figure 4:**
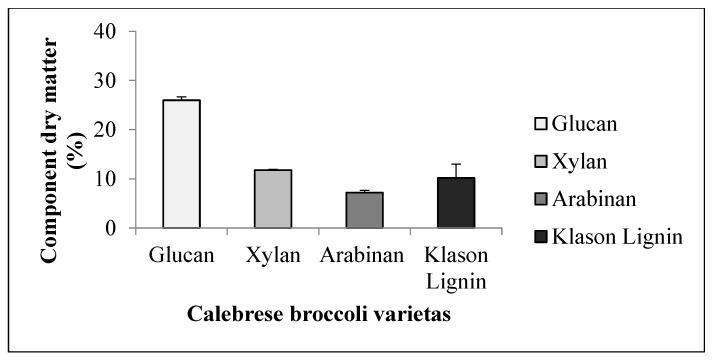
Average Klason lignin content and sugar components of broccoli (+SD).

The glucan content (25.97 *w/w*%) was less than those for the first two species. The xylan content was 11.77 *w/w*%, while the arabinan content was 7.2 *w/w*%. The value of 10.19 *w/w*% of Klason lignin (Figure 4) was lower than that of alfalfa and soy, which is also due to the fact that, in the case of broccoli, it is the leaf and the petiole that can be a source of fibre, being left as byproducts after the utilisation of the inflorescence for food.

### 3.3. Total Phenol Content (TPC), Total Flavonoid Content (TFC), Total Protein Content (TP)

#### 3.3.1. Total Phenol Content (TPC) and Total Flavonoid Content (TFC)

In the case of alfalfa, the TPC and TFC were determined by the variety and the harvest time together, which is caused by the interaction, and an Eta^2^ of 0.8 or higher was found for each. For the TPC, we detected that the highest values for each variety were measured during the first harvest. The highest values were observed during the first harvest of Plato (57.61 µg GAE/g) and Olimpia (51.86 µg GAE/g). The third harvest of Dimitra (DIM3) produced the lowest value with 32.17 µg GAE/g, which was significantly different. For the TFC, the highest value (5.45 μg rutin eq/g) was observed in the third harvest of Olimpia, and the difference was statistically justified. The lowest value (2.75 μg rutin eq/g) was observed in the first harvest of Dimitra.

In the case of soy, the TPC and TFC were also jointly determined by the two independent variables, so interactions were also observed. From the measurements, we found that the first harvest had a higher value, with the exception of the Advisor variety. The first harvest of Pannonia kincse was significantly different, where the highest value was 51.78 µg GAE/g. The lowest value (33.68 µg GAE/g) was observed in the second harvest of Isidor. For the TFC, we measured higher values in the second harvests compared to the first harvests, with the exception of the Pannónia kincse (PK1) variety, which had the highest value (4.53 μg rutin eq/g) from the first harvest. The smallest value was in the first harvest of the Isidor variety, which was 2.69 μg rutin eq/g.

For broccoli, the TPC was 33.70 µg GAE/g and the TFC was 2.80 μg rutin eq/g. The TPC and TFC values are shown in Table 1.

#### 3.3.2. Total Protein (TP)

In alfalfa, the TP value was determined by the interaction between the variety and harvest time. In all cases, we observed that the TP value increased in each variety from the first harvest to the third harvest. The highest values were observed in the third harvest of Danubia (76.77 mg/g), Dimitra (76.26 mg/g) and Olimpia (77.06 mg/g). The lowest value was measured for the first harvest of Dimitra (36.65 mg/g). However, we also observed that the Plato variety had high values in all three harvests and showed the least variation between the harvests.

It is also true for soy that harvest time and variety together determine the value of the dependent variable (TP). The highest protein value was measured in the first harvest of Advisor (45.35 mg/g), where the difference was significant. The lowest value was observed in the second harvest of Bólyi, where the value obtained was 32.28 mg/g. For broccoli, the protein content was 36.08 mg/g. The TP values are shown in Table 1.

## 4. Discussion

Circular farming is of high importance nowadays, the idea being to use all the products of production, with as little waste as possible or zero waste. A good way to achieve this is green biorefining, where green biomass is wet fractionated. Wet-fractionation methods result in fibre-rich fractions. Such fractions were evaluated in this study, including the comparison of three farm crops. Alfalfa and forage soy are well-known in green biorefinery technology as dedicated species, and broccoli was included because of the high amount of byproduct left after harvesting. The quality of the green fractionated fibre from the three crop species showed marked differences.

For soybeans, as well as for alfalfa, in most cases there was an interaction between harvest time and variety, with the exception of fibre yield and Klason lignin content. No significant differences in fibre yields were observed.

In the case of alfalfa, weather conditions have a significant influence on the nutritional value [34]. The maturity of the species significantly affects the chemical composition of the cell wall, as lignin increases with aging, which increases the proportion of indigestible fibres [35]. Xu et al. [36] measured the lignocellulosic constituents, where Klason lignin, glucan, xylan and arabinan were 15.2 *w/w*%, 30.2 *w/w*%, 9.7 *w/w*% and 3.8 *w/w*%., respectively. Our results are in line with those of Xu et al. [36] and quite close to those of Duncan and Schilling [37] and MacLellan et al. [38], obtaining glucan ranging from 27.6 to 32.59 and an arabinan content from 2.74 to 5.37 *w/w*%. It should be noted that we measured a higher xylan content, ranging from 11.62 to 15.34 *w/w*%, depending on the varieties and harvest times. However, the Klason lignin content (13.02 to 16.76 *w/w*%) we measured is lower compared to the range of 19.4–22.0 *w/w*% by Duncan and Schilling [37]. The development of values can be influenced by a number of factors such as varieties, climatic conditions and other abiotic and biotic factors. The results show that the Plato variety stands out from the other varieties. According to Popovic et al. [39], the weather and the time of cutting affect the protein content in the leaf and stem and the fibre content in the stem, but do not affect the fibre content in the leaf. No significant difference was observed for fibre yield. The amount of pressed fibre was about 25–30 percent of the green biomass. This is a similar value to our previous studies [40]. The protein content ranged from 36.55 to 77.6 mg/g DW. It is worth mentioning that the protein content in the third harvests was the highest for all varieties, which could be due to the rainy autumn weather. The phenol content ranged from 32.17 to 57.61 µg GAE/g DW. For the phenol content, the highest values were recorded in the first harvests.

In the case of soy, the plant parts applied to the investigation have an influence on the values of the parameters to be measured. Kovačić et al. [41] investigated lignocellulosic components in soy stems, where the glucan content was 34.09 *w/w*%, xylan was 11.4 *w/w*%, arabinan was 1.0 *w/w*% and Klason lignin was 18 *w/w*%. Compared to our results regarding fibre, the glucan (29.10–37.42 *w/w*%) and xylan (12.19–15.16 *w/w*%) values are similar, but the value is higher for arabinan (3.73–5.14 *w/w*%) and lower for Klason lignin (11.14–13.39 *w/w*%) in all varieties. However, Rambo et al. [42] measured the parameters we measured in the soy husk. The amount of glucan was 35.05 *w/w*%, xylan was 9.85 *w/w*% and arabinan was 4.64 *w/w*% in the dry matter. Based on this, we can conclude that similar values can be observed in soy husks and green biomass. It should be noted that Colletti et al. [43] measured a significantly lower Klason lignin content in soy hulls (1.64 *w/w*%). The protein content was 32.28–45.35 mg/g DW and the phenol content was 33.68–51.78 µg GAE/g DW. The Isidor variety was significantly different from the other varieties. Higher values were measured in several cases.

In broccoli production, a large amount of green biomass is lost after harvesting. This is due to the fact that the above-ground part of the flower accounts for 20 to 30 percent of the total, and there are losses in its utilisation during harvesting, storage and processing. Consequently, the remaining part is the green biomass, which is valuable because it is extremely rich in nutrients such as vitamins, minerals and dietary fibre. It is therefore advisable to use green biomass wisely [44]. The pressed fibre and arabinan contents in the broccoli were higher than in the alfalfa and soy varieties included in this study. The lignin, glucan and xylan contents, however, were slightly lower in comparison with those in the alfalfa and soy. Berndtsson et al. [45] measured 1.6–1.8 *w/w*% Klason lignin in broccoli leaves, which is much lower in comparison to the value we measured (10.16 *w/w*%). No previous studies measuring the phenolic content in pressed fibre resulting from wet fractionation were found, but Shiva and Jung-Ho [46] measured the phenolic content of whole leaves in 12 broccoli varieties. Their values differ from the values measured in our pressed fibre. This may be due to a difference in the method, less phenol remaining in the pressed fibre after wet fractionation and lignocellulosic compounds binding to these compounds and being difficult to dissolve. The protein content we measured was 36.08 mg/g DW.

## 5. Conclusions

Today, there is a clear increase in population, which is why circular farming plays an important role. The aim is essentially to use all the biomass generated by agricultural production without loss. In the present study, the pressed fibre from fresh biomass produced in green biorefining was evaluated as a byproduct. The study of fibre is important because it can serve as a feedstock for many industries, not least the food industry. Alfalfa and soybean species well-known in green biorefining were included in the study. Broccoli was also included in the study, as a significant amount of green biomass remains as a loss after flower harvesting, but it has value and is highly applicable in green biorefining.

In the present study, pressed fibre and the pressed fibre’s Klason lignin, glucan, xylan and arabinan contents were determined. We also measured the total protein, total phenol and flavonoid content of the pressed fibre. For alfalfa and soybean, different varieties and harvesting times were compared. For broccoli, one variety and harvest time was applied.

In the case of alfalfa and soy, the investigated factors (variety and harvest time) do not affect pressed fibre yield. During the wet fractionation of the alfalfa varieties, an average 3–5% higher lignin content could be detected in the pressed fibre compared to soy and broccoli. In alfalfa, the lignin content decreases in parallel with the harvest time, while it increases in soybeans. In the case of alfalfa, the variable lignin content is due to the combined effect of the two factors (harvest time and variety). Because lignin is the least digestible dietary fibre for microbes in the human body of all the components studied, it is advisable to choose varieties with lower lignin content, as well as considering the harvest.

The pressed fibres of the alfalfa varieties examined were 2–5% lower in glucan content compared to soy, while this value was 5–8% higher compared to broccoli. Of the three plants studied, broccoli had the lowest glucan content.

There was no significant difference between the xylan content of the three model plants studied. Harvest time had the opposite effect on the xylan content. It decreased with alfalfa and rose with soy. In the case of both plants, the variable xylan content was due to the combined effect of the two factors (harvest time and variety).

The content of press fibre arabinan was the highest for broccoli. This property makes it promising for use in functional foods, as arabinose inhibits the digestion of sucrose, even in small amounts, and therefore, its consumption may be beneficial for diabetic patients.

The total phenol (TPC), total flavonoid (TFC) and the total protein (TP) content of the fibre were highest for alfalfa.

Overall, our results may help with the selection of the right variety and harvesting time for further studies. Furthermore, they contribute to the knowledge that can be a basis for the incorporation of press fibre into the human diet through functional foods.

## Figures and Tables

**Table 1:** Total phenol content (TPC), total flavonoid content (TFC) and total protein (TP) in pressed fibre from different plants and varieties.

Plant Species	Variety and Harvest Time	TPC (µg GAE/g)	TFC (μg rutin eq/g)	TP (mg/g)
Alfalfa	DAN 1 DAN 2 DAN 3	44.34 ± 4.31b 42.00 ± 0.79bc 43.24 ± 1.16b	3.19 ± 0.26bc 3.77 ± 0.33bc 4.21 ± 0.32b	53.26 ± 5.04b 57.10 ± 5.92b 76.77 ± 0.45a
	DIM 1 DIM 2 DIM 3	44.94 ± 1.99b 37.08 ± 0.76bc *32.17 ± 1.45c*	*2.75 ± 0.01c* 3.82 ± 0.08bc 3.67 ± 0.43bc	*36.55 ± 3.08c* 40.63 ± 2.48c 76.26 ± 1.33a
	PLA 1 PLA 2 PLA 3	**57.61 ± 7.59a** 42.48 ± 0.83bc 41.88 ± 5.46bc	4.31 ± 0.72b 3.69 ± 0.16bc 3.66 ± 0.63bc	65.69 ± 3.70b 63.17 ± 2.83b 63.11 ± 3.45b
	OLI 1 OLI 2 OLI 3	51.86 ± 4.49a 37.47 ± 1.67bc 44.50 ± 3.49b	4.23 ± 0.44b 3.41 ± 0.22bc **5.45 ± 0.52a**	58.37 ± 3.45b 55.07 ± 2.52b **77.06 ± 5.36a**
Soy	ADV 1 ADV 2	35.92 ± 2.82bc 47.39 ± 5.34ab	2.97 ± 0.32b 3.28 ± 0.28b	**45.35 ± 5.50a** 37.36 ± 1.22ab
	BOL 1 BOL 2	44.76 ± 3.43ab 35.88 ± 1.80bc	3.10 ± 0.41b 3.13 ± 0.42b	42.37 ± 1.35ab *32.28 ± 1.85c*
	ISI 1 ISI 2	39.43 ± 1.15bc *33.68 ± 1.85c*	*2.69 ± 0.24b* 2.84 ± 0.27b	42.55 ± 1.73ab 43.53 ± 4.69ab
	PK 1 PK 2	**51.78 ± 1.74a** 42.94 ± 2.38b	**4.53 ± 0.60a** 3.45 ± 0.41ab	34.97 ± 1.85c 34.36 ± 1.66c
Broccoli	Calebrese	33.70 ± 2.20	2.80 ± 0.31	36.08 ± 2.70

Results are expressed as gallic acid equivalents (μg GAE/g DW) (*n* = 3) +SD for TPC, and as rutin equivalents (μg rutin eq/g DW) (*n* = 3) +SD for TFC. For TP, results are expressed as mg/g DW (*n* = 3) +SD. The bold part indicates the highest value; the italic part indicates the lowest value. Different letters in the columns refer to significant differences (*p* ≤ 0.05).

## Data Availability

Data are contained within the article.

## References

[1] Zhang J., Cai D., Qin Y., Liu D., Zhao X. (2020). High Value-added Monomer Chemicals and Functional Bio-based Materials Derived from Polymeric Components of Lignocellulose by Organosolv Fractionation. Biofuels Bioprod. Bioref..

[2] Ontañon O.M., Ghio S., de Villegas R.M.D., Garrido M.M., Talia P.M., Fehér C., Campos E. (2019). A Thermostable GH8 Endoglucanase of Enterobacter Sp. R1 Is Suitable for β-Glucan Deconstruction. Food Chem..

[3] Kamm B., Schönicke P., Hille C. (2016). Green Biorefinery—Industrial Implementation. Food Chem..

[4] Xiu S., Shahbazi A. (2015). Development of Green Biorefinery for Biomass Utilization: A Review. Trends Renew. Energy.

[5] Paul S., Dutta A. (2018). Challenges and Opportunities of Lignocellulosic Biomass for Anaerobic Digestion. Resour. Conserv. Recycl..

[6] Pang C.H., Gaddipatti S., Tucker G., Lester E., Wu T. (2014). Relationship between Thermal Behaviour of Lignocellulosic Components and Properties of Biomass. Bioresour. Technol..

[7] Kirk T.K., Obst J.R. (1988). Lignin Determination. Methods in Enzymology.

[8] Sluiter A., Hames B., Ruiz R., Scarlata C., Sluiter J., Templeton D., Crocker D. (2008). Determination of Structural Carbohydrates and Lignin in Biomass: Laboratory Analytical Procedure (LAP).

[9] Cho S., Prosky L., Dreher M.L., Complex Carbohydrates in Foods (1999). Food Science and Technology.

[10] Tungland B.C., Meyer D. (2002). Nondigestible Oligo- and Polysaccharides (Dietary Fiber): Their Physiology and Role in Human Health and Food. Compr. Rev. Food Sci. Food Saf..

[11] James S.L., Muir J.G., Curtis S.L., Gibson P.R. (2003). Dietary Fibre: A Roughage Guide: Dietary Fibre. Intern. Med. J..

[12] Mudgil D., Barak S. (2013). Composition, Properties and Health Benefits of Indigestible Carbohydrate Polymers as Dietary Fiber: A Review. Int. J. Biol. Macromol..

[13] Csatári G., Kovács S. (2022). Dietary Fibre Prevalence and Its Role in Human Nutrition. Acta Agrar. Debr..

[14] McDougall G.J., Morrison I.M., Stewart D., Hillman J.R. (1996). Plant Cell Walls as Dietary Fibre: Range, Structure, Processing and Function. J. Sci. Food Agric..

[15] Šebečić B., Dragojević I.V., Vitali D., Hečimović M., Dragičević I. (2007). Raw Materials in Fibre Enriched Biscuits Production as Source of Total Phenols. Agric. Conspec. Sci..

[16] Murray A., Skene K., Haynes K. (2017). The Circular Economy: An Interdisciplinary Exploration of the Concept and Application in a Global Context. J. Bus. Ethics.

[17] Toop T.A., Ward S., Oldfield T., Hull M., Kirby M.E., Theodorou M.K. (2017). AgroCycle—Developing a Circular Economy in Agriculture. Energy Procedia.

[18] Geisendorf S., Pietrulla F. (2018). The Circular Economy and Circular Economic Concepts—A Literature Analysis and Redefinition. Thunderbird Int. Bus. Rev..

[19] Van Ree R., Sanders J., Bakker R., Blaauw R., Zwart R., Van Der Drift B. (2011). Biofuel-Driven Biorefineries for the Co-Production of Transportation Fuels and Added-Value Products. Handbook of Biofuels Production.

[20] Velvizhi G., Balakumar K., Shetti N.P., Ahmad E., Kishore Pant K., Aminabhavi T.M. (2022). Integrated Biorefinery Processes for Conversion of Lignocellulosic Biomass to Value Added Materials: Paving a Path towards Circular Economy. Bioresour. Technol..

[21] Damborg V.K., Stødkilde L., Jensen S.K., Weisbjerg M.R. (2018). Protein Value and Degradation Characteristics of Pulp Fibre Fractions from Screw Pressed Grass, Clover, and Lucerne. Anim. Feed. Sci. Technol..

[22] Damborg V.K., Jensen S.K., Weisbjerg M.R., Adamsen A.P., Stødkilde L. (2020). Screw-Pressed Fractions from Green Forages as Animal Feed: Chemical Composition and Mass Balances. Anim. Feed. Sci. Technol..

[23] Stødkilde L., Lashkari S., Eriksen J., Jensen S.K. (2021). Enhancing Protein Recovery in Green Biorefineries through Selection of Plant Species and Time of Harvest. Anim. Feed. Sci. Technol..

[24] Zhou F., Hansen M., Hobley T.J., Jensen P.R. (2022). Valorization of Green Biomass: Alfalfa Pulp as a Substrate for Oyster Mushroom Cultivation. Foods.

[25] Fehér A., Fehér C., Rozbach M., Rácz G., Fekete M., Hegedűs L., Barta Z. (2018). Treatments of Lignocellulosic Hydrolysates and Continuous-Flow Hydrogenation of Xylose to Xylitol. Chem. Eng. Technol..

[26] Pol K., Mars M. (2021). L-Arabinose and D-Xylose: Sweet Pentoses That May Reduce Postprandial Glucose and Insulin Responses. Food Nutr. Res..

[27] Hollman P.C. (2001). Evidence for Health Benefits of Plant Phenols: Local or Systemic Effects?. J. Sci. Food Agric..

[28] Ghasemzadeh A., Ghasemzadeh N. (2011). Flavonoids and Phenolic Acids: Role and Biochemical Activity in Plants and Human. J. Med. Plants Res..

[29] Garlapati V.K., Chandel A.K., Kumar S.P.J., Sharma S., Sevda S., Ingle A.P., Pant D. (2020). Circular Economy Aspects of Lignin: Towards a Lignocellulose Biorefinery. Renew. Sustain. Energy Rev..

[30] Singleton V.L., Rossi J.A. (1965). Colorimetry of Total Phenolics with Phosphomolybdic-Phosphotungstic Acid Reagents. Am. J. Enol. Vitic..

[31] Kim D.-O., Chun O.K., Kim Y.J., Moon H.-Y., Lee C.Y. (2003). Quantification of Polyphenolics and Their Antioxidant Capacity in Fresh Plums. J. Agric. Food Chem..

[32] Bradford M.M. (1976). A Rapid and Sensitive Method for the Quantitation of Microgram Quantities of Protein Utilizing the Principle of Protein-Dye Binding. Anal. Biochem.

[33] Abdi H., Williams L. (2021). Tukey’s Honestly Signiflcant Difierence (HSD) Test. Encycl. Res. Des..

[34] Brink G., Hall M., Shewmaker G., Undersander D., Martin N., Walgenbach R. (2010). Changes in Alfalfa Yield and Nutritive Value within Individual Harvest Periods. Agron. J..

[35] Palmonari A., Fustini M., Canestrari G., Grilli E., Formigoni A. (2014). Influence of Maturity on Alfalfa Hay Nutritional Fractions and Indigestible Fiber Content. J. Dairy Sci..

[36] Xu N., Zhang W., Ren S., Liu F., Zhao C., Liao H., Xu Z., Huang J., Li Q., Tu Y. (2012). Hemicelluloses Negatively Affect Lignocellulose Crystallinity for High Biomass Digestibility under NaOH and H_2_SO_4_ Pretreatments in Miscanthus. Biotechnol. Biofuels.

[37] Duncan S.M., Schilling J.S. (2010). Carbohydrate-Hydrolyzing Enzyme Ratios during Fungal Degradation of Woody and Non-Woody Lignocellulose Substrates. Enzym. Microb. Technol..

[38] MacLellan J., Chen R., Yue Z., Kraemer R., Liu Y., Liao W. (2017). Effects of Protein and Lignin on Cellulose and Xylan Anaylses of Lignocellulosic Biomass. J. Integr. Agric..

[39] Popovic S., Grljusic S., Cupic T., Tucak M., Stjepanovic M. (2001). Protein and Fiber Contents in Alfalfa Leaves and Stems. Proceedings of the Options Méditerranéennes. Série A: Séminaires Méditerranéens (CIHEAM).

[40] Csatári G., Eged B., Görögh P., Fári M., Kovács S. Comparative Evaluation of Fibers Obtained by Wet Fractionation from Different Plants (*Medicago sativa* L., *Glycine max* L., *Brassica Oleracea Conv. Botrytis var. Italica*). Proceedings of the LXIII. Georgikon Napok: Agrár-Környezetgazdaságunk a Járványok és a Környezeti Kihívások Árnyékában.

[41] Kovačić Đ., Kralik D., Rupcic S., Jovicic D., Spajic R., Tisma M. (2017). Soybean Straw, Corn Stover and Sunflower Stalk as Possible Substrates for Biogas Production in Croatia: A Review. Chem. Biochem. Eng. Q..

[42] Rambo M.K.D., Schmidt F.L., Ferreira M.M.C. (2015). Analysis of the Lignocellulosic Components of Biomass Residues for Biorefinery Opportunities. Talanta.

[43] Colletti A.C., Delgado J.F., Cabezas D.M., Wagner J.R., Porfiri M.C. (2020). Soybean Hull Insoluble Polysaccharides: Improvements of Its Physicochemical Properties Through High Pressure Homogenization. Food Biophys..

[44] Domínguez-Perles R., Martínez-Ballesta M.C., Carvajal M., García-Viguera C., Moreno D.A. (2010). Broccoli-Derived By-Products—A Promising Source of Bioactive Ingredients. J. Food Sci..

[45] Berndtsson E., Andersson R., Johansson E., Olsson M.E. (2020). Side Streams of Broccoli Leaves: A Climate Smart and Healthy Food Ingredient. Int. J. Environ. Res. Public Health.

[46] Shiva R.B., Jung-Ho K. (2014). Seasonal Variation in Phytochemicals and Antioxidant Activities in Different Tissues of Various Broccoli Cultivars. Afr. J. Biotechnol..

